# HOTAIRM1 Maintained the Malignant Phenotype of tMSCs Transformed by GSCs via E2F7 by Binding to FUS

**DOI:** 10.1155/2022/7734413

**Published:** 2022-05-09

**Authors:** Liang Liu, Yanling Zhou, Xuchen Dong, Suwen Li, Shan Cheng, Haoran Li, Yongdong Li, Jiaqi Yuan, Liping Wang, Jun Dong

**Affiliations:** Department of Neurosurgery, Second Affiliated Hospital of Soochow University, Suzhou 215004, China

## Abstract

**Objective:**

Mesenchymal stromal/stem cells (MSCs) are an important part of the glioma microenvironment and are involved in the malignant progression of glioma. In our previous study, we showed that MSCs can be induced to a malignant phenotype (tMSCs) by glioma stem cells (GSCs) in the microenvironment. However, the potential mechanism by which tMSCs maintain their malignant phenotype after malignant transformation has not been fully clarified.

**Methods:**

The expression of HOTAIRM1, FUS, and E2F7 was detected by qRT-PCR. Clone formation, EdU, and Transwell assay were used to explore the role of HOTAIRM1, FUS, and E2F7 on the proliferation, migration, and invasion of tMSCs. Bioinformatics analysis and RNA immunoprecipitation were used to explore the relation among HOTAIRM1, FUS, and E2F7.

**Results:**

HOTAIRM1 was upregulated in tMSCs compared with MSCs. Loss- and gain-of-function assays showed that HOTAIRM1 promoted the proliferation, migration, and invasion of tMSCs. qRT-PCR and functional assays revealed that E2F7 might be the downstream target of HOTAIRM1. A further study of the mechanism showed that HOTAIRM1 could bind to FUS, an RNA-binding protein (RBP), and thus regulate E2F7, which could promote the malignant phenotype of tMSCs.

**Conclusion:**

Our study revealed that the HOTAIRM1/FUS/E2F7 axis is involved in the malignant progression of tMSCs transformed by GSCs in the glioma microenvironment and may function as a novel target for glioma therapy.

## 1. Introduction

Glioma is the most common primary malignant tumour in the adult central nervous system and is characterized by a short survival and high recurrence [1, 2]. Due to its special location and characteristics, glioma has become a great challenge in neuro-oncology [3]. Therefore, in-depth exploration of the molecular mechanism of glioma progression and the search for new diagnostic and treatment targets have important theoretical value for glioma therapy.

In glioma tissue, in addition to glioma stem cells (GSCs) and their progeny tumour cells, there are many kinds of stromal cells that constitute the microenvironment of glioma [4, 5]. Existing studies have shown that these stromal cells in the glioma microenvironment play an important role in the progression of glioma [6]. On the one hand, GSCs and their progeny cells can lead to malignant transformation of these stromal cells; on the other hand, these malignantly transformed stromal cells can promote the development of glioma cells by remodifying the microenvironment of glioma [7–10]. Mesenchymal stromal/stem cells (MSCs) are pluripotent stem cells that have the characteristics of multidirectional differentiation potential, immune regulation, and haematopoietic maintenance [11, 12]. Studies have shown that MSCs can directly migrate into glioma tissues and become components of the glioma microenvironment [13, 14]. Because MSCs can specifically target tumour cells, there are current studies indicating that MSCs can be used as an ideal carrier for tumour-targeted therapy [15]. At present, the role of MSCs in the progression of glioma is still controversial. Some scholars believe that MSCs can promote the progression of glioma, while others believe that they exert an inhibitory effect [16]. In our previous study, with the help of orthotopic models of GSC-MSC interactions, we showed that the MSCs that migrated into the glioma environment were malignantly transformed by GSCs, which might be related to the malignant progression of gliomas [17]. However, the potential mechanism by which malignantly transformed MSCs (tMSCs) maintain their malignant phenotype after malignant transformation has never been reported.

Long noncoding RNAs (lncRNAs) are an important part of noncoding RNAs (ncRNAs). Although they were initially identified as “noise” during transcription, researchers have gradually realized that they are specific signals of the cell state that can identify tumour cells and even become novel targets for tumour therapy [18, 19]. Studies have also reported that lncRNAs are dysregulated in glioma and related to glioma occurrence and development [17, 20]; however, the underlying mechanism has not been clarified. HOXA transcript antisense RNA myeloid-specific 1 (*HOTAIRM1*) is a lncRNA that was first reported in 2009. To date, it has been shown to be related to many kinds of human diseases, such as prostate cancer [21], anaplastic thyroid cancer [22], and acute myeloid leukaemia cells [23]. In glioma, studies have shown that *HOTAIRM1* can maintain the tumourigenesis of GSCs and promote the proliferation, migration, and invasion of glioma cells [24–26]; however, its role in tMSCs in the glioma environment has never been studied.

In the current study, we found that *HOTAIRM1* was upregulated in tMSCs compared with MSCs. The results of this mechanistic study showed that *HOTAIRM1* could bind to FUS, an RNA-binding protein (RBP), and thus regulate E2F7, which could promote the malignant phenotype of tMSCs. Collectively, this study revealed that the *HOTAIRM1*/FUS/E2F7 axis is involved in the maintenance of the malignant phenotype of tMSCs and may function as a novel target for glioma therapy.

## 2. Methods and Materials

### 2.1. Cell Culture

The cells used in this study, including normal murine MSCs and transformed MSCs (tMSC1, tMSC2, and tMSC3), were constructed and validated as described in our previous studies [17]. Cells were cultured in DMEM/F12 (HyClone, USA, catalog No. SH30023.01B) supplemented with 10% FBS (ScienCell, LA, USA, catalog No. #0500) and maintained in an incubator (5% CO_2_ at 37°C).

### 2.2. Cell Transfection

Short hairpin RNAs (shRNAs) targeting *HOTAIRM1*, *FUS*, and *E2F7* were all provided by GenePharma (Shanghai, China). For cell transfection, serum-free medium (500 *μ*l), plasmid (4 *μ*g), and lipofectamine 3000 (10 *μ*l, Invitrogen, CA, USA) were mixed and left at room temperature for 20 min to form the RNA/lipo mixture. Subsequently, the mixture was added to the serum-free medium cultured cells. After 6 h, the used medium was replaced with fresh complete medium, and the culture was continued for 48 h. For the construction of stable transfected cell lines, virus solution (100 *μ*l) was added to complete medium (1 ml), and then polybrene with a concentration of 10 mg/ml (0.5 *μ*l) was added and mix (final polybrene concentration is 5 *μ*g/ml). When the cell density is about 80%, the used medium is discarded, virus infection mixture (0.25 ml) is added to each well of the 24-well plate. After 24 h of incubation, the virus infection mixture was discarded, and fresh complete medium was added to continue the culture for 24 h. Cells were passaged when the cells grew to 90% density. After the cells adhered, complete medium containing puromycin (2 *μ*g/ml) was added to each well for selection.

### 2.3. Reverse Transcription Quantitative Polymerase Chain Reaction (qRT-PCR)

Total RNA was isolated with TRIzol reagent (Invitrogen, catalog No. 15596-026) according to the protocol provided by the manufacturer. The RNA quality was assessed using a NanoDrop 2000 spectrometer (Thermo Fisher Scientific, MA, USA). The PrimeScript™ RT reagent Kit (Takara, Japan, catalog No. RR037A) was used to conduct reverse transcription reactions. qRT-PCR was used to detect the relative expression of *HOTAIRM1*, *FUS*, and *E2F7* by using the SYBR Green Kit (Thermo Fisher Scientific, catalog No. 00920335) and ABI 7300 System (Applied Biosystems, Foster City, CA, USA). *GAPDH* was used for normalization. The 2^–*ΔΔ*Ct^ method was used to analyse differences. The primers used in this study are shown in Table 1.

### 2.4. Western Blot Analysis

Total protein from cells was isolated with RIPA buffer (KenGEN, Shanghai, China, catalog No. KGP250/KGP2100), separated by SDS-PAGE (10%, Beyotime, Shanghai, China, catalog No. P0690), and transferred to PVDF membranes. After being cultured with skimmed milk (5%), membranes were incubated with primary antibodies against FUS (Proteintech, USA, catalog No. 11570-1-AP), E2F7 (Proteintech, catalog No. 24489-1-AP), and GAPDH (Proteintech, catalog No. 60004-1-Ig) overnight. Next, the membranes were incubated with horseradish peroxidase-tagged secondary antibody for 2 hours. The bands were then determined using an Image Quant LAS 4000 mini (GE, USA).

### 2.5. RNA Immunoprecipitation (RIP) Assay

RNA immunoprecipitation (RIP) experiments were performed with a Magna RIP™ RNA-Binding Protein Immunoprecipitation Kit (Millipore, USA, catalog No. 17-700). Cells were harvested and lysed in RIP lysis buffer. Next, the cell extract (100 *μ*l) was incubated with RIP buffer containing magnetic beads conjugated to the indicated antibodies at 4°C for 6 hours. Then, the beads were washed with washing buffer, and immunoprecipitated RNA was purified and analysed by qRT-PCR.

### 2.6. Nuclear and Cytoplasmic RNA Fraction Isolation

Nuclear and cytoplasmic RNA were isolated from each fraction using a Nuclear/Cytosol Fractionation Kit (BioVision, San Francisco, USA, Cat. No. XY-K266-25) following the manufacturer's instructions. U6 and 18S were used as a nuclear control and cytoplasmic control, respectively.

### 2.7. Colony Formation Assays

Colony formation assays were conducted as described in our previous study. Namely, cells cultured in DMEM/F12 supplemented with 10% FBS were added to culture dishes (60 mm). After culturing in an incubator containing 5% CO_2_ at 37°C for 14 days, the colonies were fixed with paraformaldehyde (4%), stained with crystal violet, and visualized under a dissection microscope (Olympus). Cell colonies consisting of >50 cells were counted and used for comparisons of colony formation ability.

### 2.8. 5-Ethynyl-20-deoxyuridine (EdU) Assay

The EdU assay was conducted as described in our previous study. Briefly, cells were added to a 96-well plate. Ten hours later, EdU medium (RiboBio, Guangzhou, China, catalog No. C00003) was added to the plate and cultured for 2 h. After being fixed with paraformaldehyde (4%) and permeabilized with Triton X-100 (0.4%, KeyGen, Shanghai, China, catalog No. KGF001), cell nuclei were stained with Hoechst33342 (Beyotime, Shanghai, China, catalog No. C1002). Finally, a fluorescence microscope (Olympus, Tokyo, Japan) was used to observe the EdU-positive cells. EdU incorporation rate was expressed as the ratio of EdU-positive cells (red cells) to total Hoechst33342-positive cells (blue cells).

### 2.9. Transwell Assay

Transwell assays were performed as described in our previous study. Cells in the logarithmic growth phase cultured in DMEM/F12 without FBS were added to the upper chamber (Millipore, MA, USA). Then, 600 *μ*l DMEM/F12 supplemented with 10% FBS was added to the bottom chamber. Thirty-six hours later, cells still in the upper chamber were removed, and cells on the basement membrane were fixed with paraformaldehyde (4%) and then stained with crystal violet. The numbers of migrating and invading cells were counted with an inverted microscope (Olympus, Tokyo, Japan). The difference between migration and invasion was that for the invasion assay, the basement membrane was precoated with Matrigel (Matrigel : DMEM = 1 : 9, BD, USA, catalog No. 356254).

### 2.10. Immunohistochemistry

The tissue was embedded in paraffin and then made into sections (5 *μ*m). Then, the sections were deparaffinized and rehydrated. After antigen retrieval, sections were blocked with 5% goat serum and then incubated with the following primary antibody (Proteintech, catalog No. 27309-1-AP) overnight at 4°C and finally stained with horseradish peroxidase- (HRP-) conjugated secondary antibody (Proteintech, catalog No. PR30009). Images were obtained using an upright metallurgical microscope (Olympus). The number of positive cells for the marker was expressed relative to the total number of cells.

### 2.11. Tumour Xenograft in Nude Mice

Ten BALB/c female nude mice aged 4-5 weeks were purchased from Beijing Laboratory Animal Center (Beijing, China). tMSC1 (1,000,000) transfected with sh-RNA targeting HOTAIRM1 or control was subcutaneously injected into the right shoulder of nude mice (5 per group). Twenty-seven days later, the mice were euthanized, and the tumours were removed. The weight, length, and width of the tumours were measured.

### 2.12. Statistical Analysis

The data were analysed by GraphPad 8.0 and presented as the mean ± standard deviation. Student's *t*-test was used for two-group comparisons. For comparisons among more than two groups, the Wilcoxon test and one-way ANOVA were used for nonparametric and parametric data. *P* < 0.05 indicates statistical significance. All assays were performed three times independently.

## 3. Results

### 3.1. HOTAIRM1 Was Upregulated in tMSCs and Promoted the Proliferation, Migration, and Invasion of tMSCs

**Figure 1:**
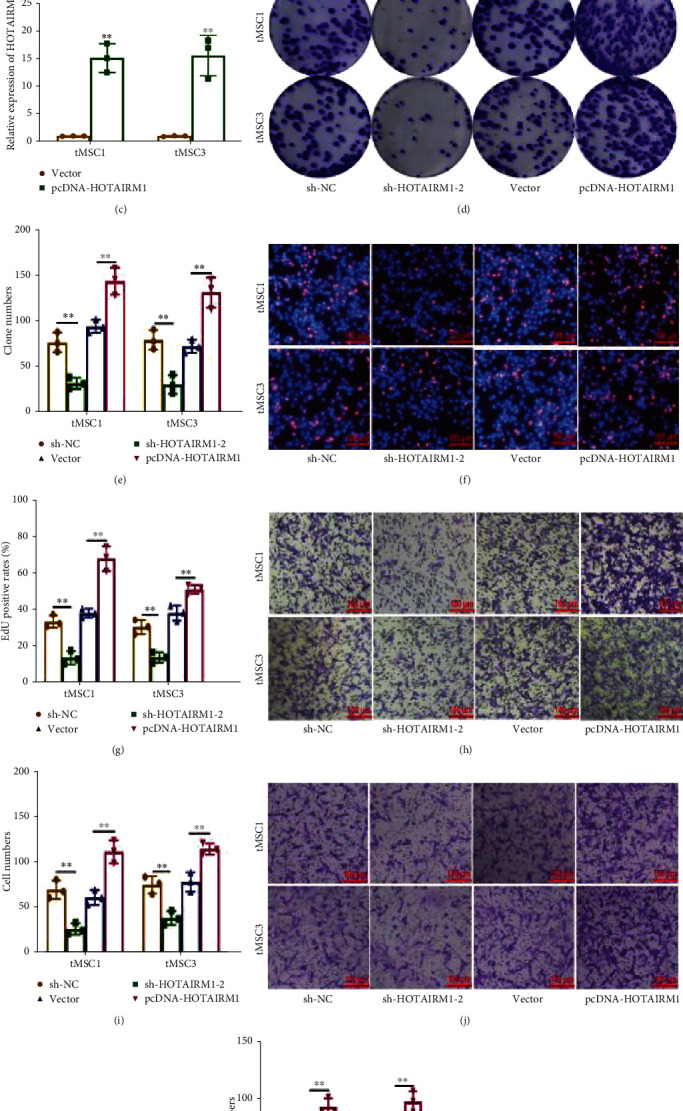
HOTAIRM1 is upregulated in tMSCs and promotes the proliferation, migration, and invasion of tMSCs. (a) qRT-PCR analysis of HOTAIRM1 expression in normal mesenchymal stromal/stem cells (MSCs) and transformed mesenchymal stromal/stem cells (tMSCs). (b) qRT-PCR analysis of HOTAIRM1 expression in tMSC1 and tMSC3 cells transfected with control (sh-NC), sh-HOTAIRM1-1, and sh-HOTAIRM1-2. (c) qRT-PCR analysis of HOTAIRM1 expression in tMSC1 and tMSC3 cells transfected with empty vector (Vector) and pcDNA-HOTAIRM1. (d, e) Colony formation assays were performed to detect the proliferation of tMSC1 and tMSC3 cells with different expression levels of HOTAIRM1. (f–g) EdU assays were used to determine the proliferation of tMSC1 and tMSC3 cells with different expression levels of HOTAIRM1. (h, i) Transwell assays were performed to detect changes in migratory abilities of tMSC1 and tMSC3 cells with different expression levels of HOTAIRM1. (j, k) Transwell assays were performed to detect changes in invasion abilities of tMSC1 and tMSC3 cells with different expression levels of HOTAIRM1. ∗∗ means *P* < 0.01.

First, the expression levels of *HOTAIRM1* in tMSCs were detected by qRT-RCR, and the results showed that compared with normal MSCs, HOTAIRM1 was overexpressed in tMSCs, especially in tMSC1 and tMSC3 (Figure 1(a)). To assess the role of *HOTAIRM1* in tMSCs, HOTAIRM1 siRNA, HOTAORM1 overexpression plasmid, and the corresponding control were synthesized and transfected into tMSC1 and tMSC3 cells (Figures 1(b) - 1(c)). Colony formation and EdU assays showed that silencing *HOTAIRM1* suppressed proliferation; however, overexpressing *HOTAIRM1* promoted the proliferation ability of tMSC1 and tMSC3 (Figures 1(d)–1(g)). Transwell assays were performed to detect changes in the migratory and invasion abilities of tMSC1 and tMSC3 cells. The results showed that *HOTAIRM1* siRNA inhibited the migratory and invasion abilities, whereas the *HOTAIRM1* overexpression plasmid accelerated the migration and invasion of tMSC1 and tMSC3 cells (Figures 1(h)–1(k)). These results demonstrate that *HOTAIRM1* is conducive to maintenance of the malignant phenotype of tMSCs.

### 3.2. HOTAIRM1 Promotes the Growth of Tumours Formed by tMSCs

**Figure 2:**
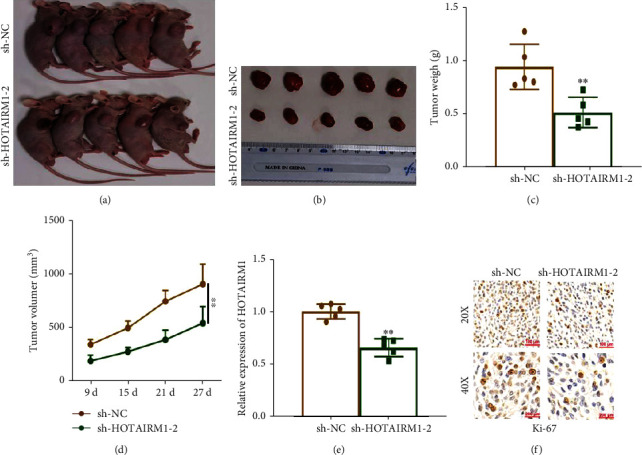
HOTAIRM1 promotes the growth of tumours formed by tMSCs. (a, b) Tumours formed by tMSCs in nude mice. (c) Tumour weights were measured after tumour removal. (d) Tumour volumes were calculated after tumour removal. (e) qRT-PCR was performed to detect the average expression of HOTAIRM1 in xenograft tumours. (f) The tumour sections were subjected to IHC staining using an antibody against Ki-67. ∗∗ means *P* < 0.01.

To investigate the role of *HOTAIRM1* in the growth of tumours formed by tMSCs, sh-HOTAIRM1 and the control were transfected into tMSC1 cells. Next, the transfected tMSCs were subcutaneously injected into the right shoulder of nude mice. After 27 days, the formed tumours were removed (Figures 2(a) - 2(b)). The data suggested that the weight and volume of the tumours formed by sh-HOTAIRM1-transfected tMSC1 cells were decreased compared with the control group (Figures 2(c) - 2(d)). The expression level of *HOTAIRM1* in tumours formed by sh-HOTAIRM1-transfected tMSC1 cells was lower than in the control group (Figure 2(e)). In addition, immunohistochemical analysis indicated that the tumours developed by sh-HOTAIRM1-transfected tMSC1 cells showed a lower density of Ki-67 than in the control group (Figure 2(f)). These data illustrate that *HOTAIRM1* promotes the tumourigenicity of tMSC1 *in vivo*.

### 3.3. HOTAIRM1 Can Bind to FUS

**Figure 3:**
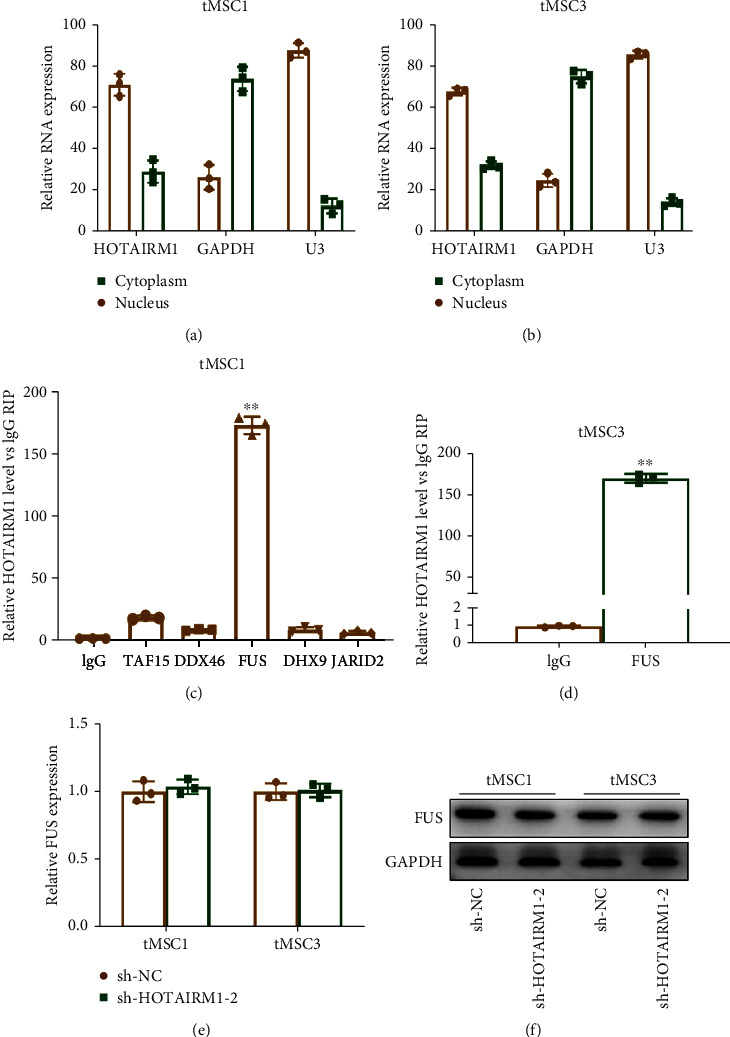
HOTAIRM1 can bind to FUS. (a, b) qRT-PCR was used to verify the subcellular localization of HOTAIRM1 in tMSC1 and tMSC3 cells. Change in E2Fs after HOTAIRM1 knockdown in tMSC1 and tMSC3 cells. (c) In tMSC1, RIP experiments were performed for the indicated RBPs, and coprecipitated RNA was subjected to qRT-PCR for HOTAIRM1. (d) RIP experiments were performed for FUS, and the coprecipitated RNA was subjected to qRT-PCR for HOTAIRM1 in tMSC3. (e, f) qRT-qPCR and Western blotting were performed to detect FUS mRNA and protein levels after HOTAIRM1 knockdown in tMSC1 and tMSC3 cells. ∗∗ means *P* < 0.01.

To clarify the mechanism by which *HOTAIRM1* regulates the malignant phenotype of tMSCs, subcellular localization of *HOTAIRM1* was detected by qRT-PCR. The results showed that *HOTAIRM1* was mainly existed in nucleus (Figures 3(a) - 3(b)) which indicated that *HOTAIRM1* plays regulatory roles via recruiting RNA-binding proteins (RBPs) [27, 28]. Next, starBase was used to search the RBPs to which *HOTAIRM1* could bind, and five RBPs were screened out. The RIP assay showed that the abundance of *HOTAIRM1* bound to FUS was the highest in tMSC1 and tMSC3 (Figures 3(c) - 3(d)). Next, qRT-PCR and Western blotting were used to explore the effect of *HOTAIRM1* on FUS, and the results indicated that silencing of HOTAIRM1 could not regulate FUS at either the mRNA or protein level (Figures 3(e) - 3(f)). The role of FUS in the proliferation, migration, and invasion of tMSC1 and tMSC3 cells is shown in Figure S1. These findings suggested that *HOTAIRM1* could bind to FUS, thus regulating the malignant phenotype of tMSCs.

### 3.4. HOTAIRM1 Promotes the Proliferation, Migration, and Invasion of tMSCs by Regulating E2F7

**Figure 4:**
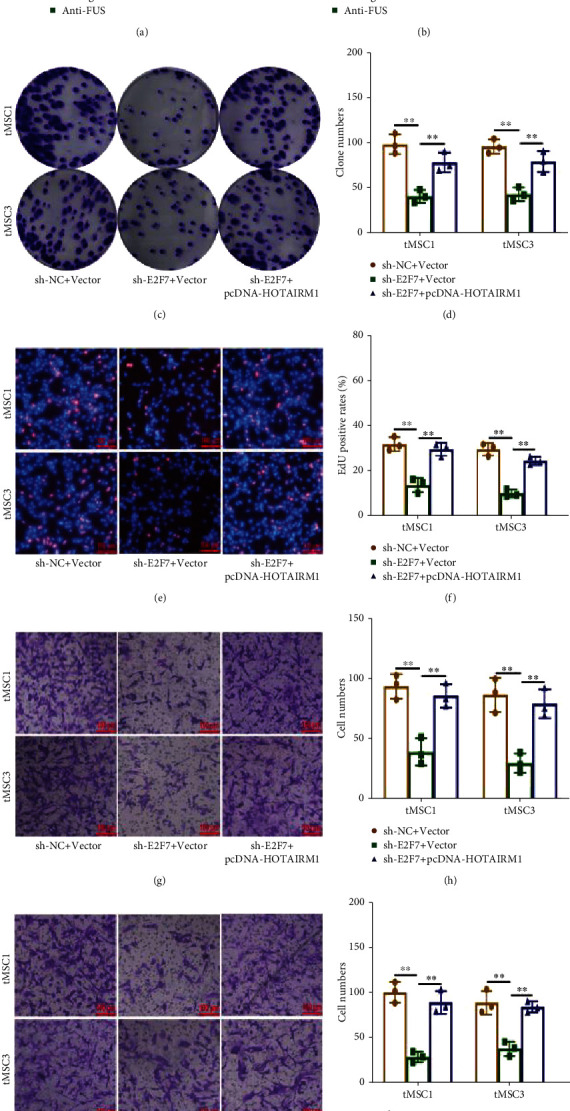
HOTAIRM1 promotes the proliferation, migration, and invasion of tMSCs by regulating E2F7. (a, b) In tMSC1 and tMSC3, RIP experiments were performed for FUS, and coprecipitated RNA was subjected to qRT-PCR for E2Fs. (c, d) Colony formation assays were performed to evaluate the proliferation of sh-E2F7- and pcDNA-HOTAIRM1-cotransfected tMSC1 and tMSC3 cells. (e, f) EdU assays were used to determine the proliferation of sh-E2F7 and pcDNA-HOTAIRM1-cotransfected tMSC1 and tMSC3 cells. (g, h) Transwell assays were performed to detect changes in migration of sh-E2F7 and pcDNA-HOTAIRM1-cotransfected tMSC1 and tMSC3 cells. (i, j) Transwell assays were performed to detect changes in invasion of sh-E2F7 and pcDNA-HOTAIRM1-cotransfected tMSC1 and tMSC3 cells. ∗∗ means *P* < 0.01.

To investigate the mechanism by which *HOTAIRM1* maintains the malignant phenotype of tMSCs, downstream target genes are needed. E2F transcription factors (E2Fs) are a family of members that play critical roles in the progression of cancers [29, 30] and that are also the focus of our team. The data from starBase indicated that E2Fs could function as targets of FUS as well. Based on these findings, RIP assays were carried out and the results showed that E2Fs, especially E2F7, could enrich to FUS (Figures 4(a) - 4(b)). Since E2F7 was enriched most significantly by FUS, it was chosen for a further study. qRT-PCR and Western blotting showed that downregulation of FUS could decrease the expression of E2F7 (Figure S2). Colony formation assays and EdU assays showed that knockdown of E2F7 could inhibit the proliferation of tMSC1 and tMSC3, and cotransfection of the *HOTAIRM1* overexpression plasmid could rescue the inhibition of proliferation (Figures 4(c)–4(f)). Transwell assays showed that *HOTAIRM1* overexpression corrected the decrease in migration and invasion of cells caused by E2F7 sh-RNA (Figures 4(g)–4(j)). These data indicate that *HOTAIRM1* promotes the proliferation, migration, and invasion of tMSCs by regulating E2F7.

### 3.5. HOTAIRM1 Functions as an Oncogene by Regulating E2F7 by Binding to FUS

**Figure 5:**
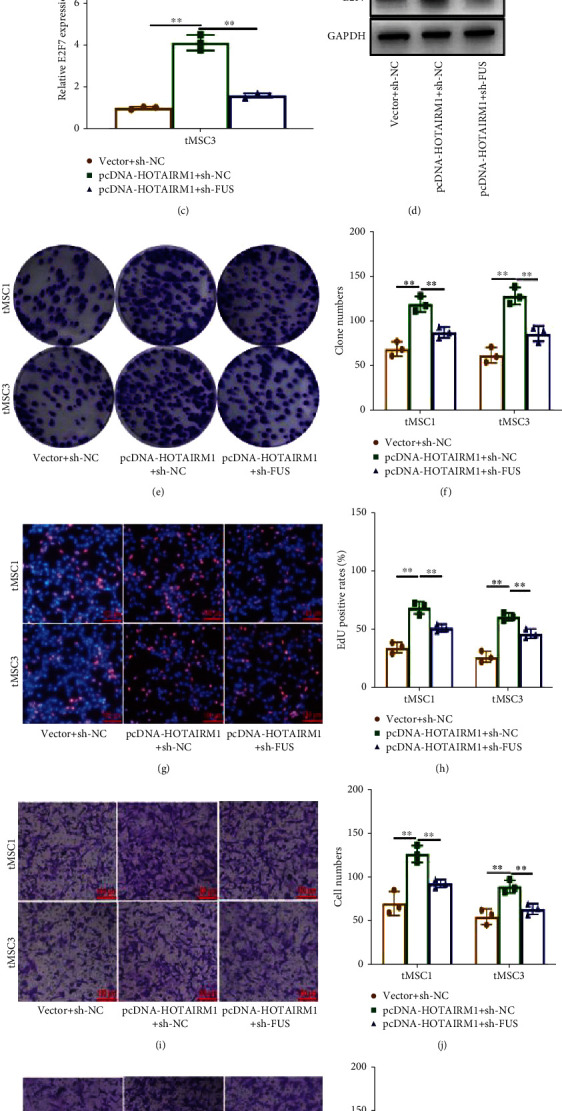
HOTAIRM1 functions as an oncogene by regulating E2F7 by binding to FUS. (a–d) qRT-PCR and Western blotting were performed to detect the expression of E2F7 in pcDNA-HOTAIRM1 and sh-FUS-cotransfected tMSC1 and tMSC3 cells. (e, f) Colony formation assays were performed to evaluate the proliferation of pcDNA-HOTAIRM1 and sh-FUS-cotransfected tMSC1 and tMSC3 cells. (g, h) EdU assays were used to determine the proliferation of pcDNA-HOTAIRM1- and sh-FUS-cotransfected tMSC1 and tMSC3 cells. (i, j) Transwell assays were performed to detect changes in migration of pcDNA-HOTAIRM1- and sh-FUS-cotransfected tMSC1 and tMSC3 cells. (k, l) Transwell assays were performed to detect changes in invasion of pcDNA-HOTAIRM1- and sh-FUS-cotransfected tMSC1 and tMSC3 cells. ∗∗ means *P* < 0.01.

To increase the rigor of the mechanism, rescue experiments were conducted. qRT-PCR and Western blot analyses showed that silencing of FUS partly eliminated the upregulation of E2F7 caused by *HOTAIRM1* overexpression (Figures 5(a)–5(d)). Colony formation and EdU assays showed that FUS knockdown decreased the promoting effect of *HOTAIRM1* on proliferation in tMSC1 and tMSC3 cells (Figures 5(e)–5(h)). Similarly, Transwell assays showed that cotransfection of FUS sh-RNA could partly counteract the promoting effect of *HOTAIRM1* on migration and invasion in tMSC1 and tMSC3 cells (Figures 5(i)–5(l)). These data illustrated that *HOTAIRM1* maintained the malignant phenotype of tMSC1 and tMSC3 by regulating E2F7 via binding to FUS.

## 4. Discussion

In recent years, investigating the interaction between tumour parenchymal cells and stromal cells in tumour progression at the tumour microenvironment level has been a hotspot in cancer research [31, 32]. The glioma microenvironment, mainly composed of GSCs, tumour cells derived from GSCs, MSCs, endothelial cells, macrophages, extracellular matrix, and metabolites, provides not only a shelter for various cells but also a place for the interaction and signal transduction between parenchymal cells and various stromal cells [33, 34]. The specific role of these stromal cells in the progression of glioma has not been determined, but there is no doubt that they are closely related to the progression of glioma.

MSCs were discovered, isolated, and identified in the 1960s by Friedenstein [14]. They have subsequently been widely studied in cancer research. Studies have indicated that MSCs can be used as vectors to deliver genes, viruses, and proteins to achieve the goal of targeting tumours [14]. However, the role of MSCs in glioma treatment is controversial. Some researchers insist that MSCs can inhibit glioma progression, because in animal experiments, MSCs were observed to migrate to glioma tissue and improve the survival of glioma-bearing mice [35]. Scholars also believe that MSCs have no effect on glioma progression or even promote glioma progression because the migration of MSCs into glioma tissue shows no effect on the tumourigenicity of mice [36]. More interestingly, some scholars have found that MSCs undergo malignant transformation after migrating into glioma tissue and have some characteristics of tumour cells. For example, Liu et al. reported that after culture with C6, the expression of wild-type p53 in MSCs was decreased, whereas mutant p53 was increased [37]. Consistent with this finding, we previously found that MSCs could be recruited and malignantly transformed by GSCs in a glioma environment [17]. However, the mechanism by which malignantly transformed MSCs maintain their malignant phenotype still merits further study.

As an important component of the transcriptome, lncRNAs have been verified to play a critical role in tumour progression. *HOTAIRM1* is a lncRNA that has been verified as an oncogene in glioma progression [38, 39]; however, whether it is involved in the maintenance of the malignant phenotype of tMSCs has never been studied. In the current study, we found that after malignant transformation by GSCs, the expression of *HOTAIRM1* was significantly upregulated. Functional assays showed that *HOTAIRM1* promoted the proliferation, migration, and invasion of tMSCs. These data suggest that *HOTAIRM1* is beneficial to the maintenance of the malignant phenotype of tMSCs, that is, to promote the progression of glioma.

To uncover the underlying mechanism, starBase was used to explore the target of *HOTAIRM1*. Combined with RIP, qRT-PCR, and Western blot, we confirmed that FUS could bind to *HOTAIRM1* which might result from the medium we searched. FUS is an RNA-binding protein that is related to DNA repair, splicing, and RNA transport [40]. In glioma, studies have indicated that FUS can interact with BACH2 (BTB domain and CNC homologue 2), thus promoting glioma progression [41]. In our study, we found that FUS not only could be recruited by HOTAIRM1 but also could regulate the proliferation, migration, and invasion of tMSC1 and tMSC3.

E2Fs are an important transcription factor family that is closely related to the cell cycle process of tumour cells and have been studied by our team for a long time. In the present study, we wondered whether *HOTAIRM1* maintained the malignant phenotype of tMSCs in an E2F-dependent manner. Thus, we detected the expression of E2Fs in *HOTAIRM1*-overexpressed tMSCs and found that E2F7, which has been reported to act as an oncogene in glioma [42–44], was also obviously increased. Functional assays showed that silencing of E2F7 could inhibit the proliferation, migration, and invasion of tMSCs, and the inhibitory effect could be restored by *HOTAIRM1*. These results suggested that E2F7, which is involved in the process of *HOTAIRM1*, maintained the malignant phenotype of tMSCs.

In conclusion, we report that *HOTAIRM1* is conducive to maintaining the malignant phenotype of tMSCs transformed by GSCs in the glioma microenvironment via E2F7 by binding to FUS and that the *HOTAIRM1*/FUS/E2F7 axis may be a potential target for glioma therapy (Figure S3).

## Figures and Tables

**Table 1:** The primers used in this study.

Name	Forward	Reverse
HOTAIRM1	GAGTCGAGACTGCCTTCTGC	ACCCCCATTTTCAGTGTGGT
FUS	GGTGGTGGAGGCAACTATGG	GTCACTTCCGCCCATGCCGC
E2F7	GATGCGTTCGTGAACTCCCTG	AGAAACTTCTGGCACAGCAGCC
GAPDH	CATCACCATCTTCCAGGAGCG	TGACCTTGCCCACAGCCTTG

## Data Availability

The data are available from the corresponding author on reasonable request.

## Supplementary Materials

Supplementary Materials Figure S1: the role of FUS in the proliferation, migration, and invasion of tMSC1 and tMSC3 cells. Figure S2: qRT-PCR and Western blotting showed that downregulation of FUS could decrease the expression of E2F7. Figure S3: the schematic overview of the HOTAIRM1/FUS/E2F7 in the malignant transformation.
