# Supplementary material for: HOTAIRM1 Maintained the Malignant Phenotype of tMSCs Transformed by GSCs via E2F7 by Binding to FUS
Source: J Oncol. 2022 May 9;2022:7734413. doi: 10.1155/2022/7734413 (PMC9110228; doi:10.1155/2022/7734413)
Supplement: Supplementary Materials — Figure S1: the role of FUS in the proliferation, migration, and invasion of tMSC1 and tMSC3 cells. Figure S2: qRT-PCR and Western blotting showed that downregulation of FUS could decrease the expression of E2F7. Figure S3: the schematic overview of the HOTAIRM1/FUS/E2F7 in the malignant transformation. [file 7734413.f1.docx]

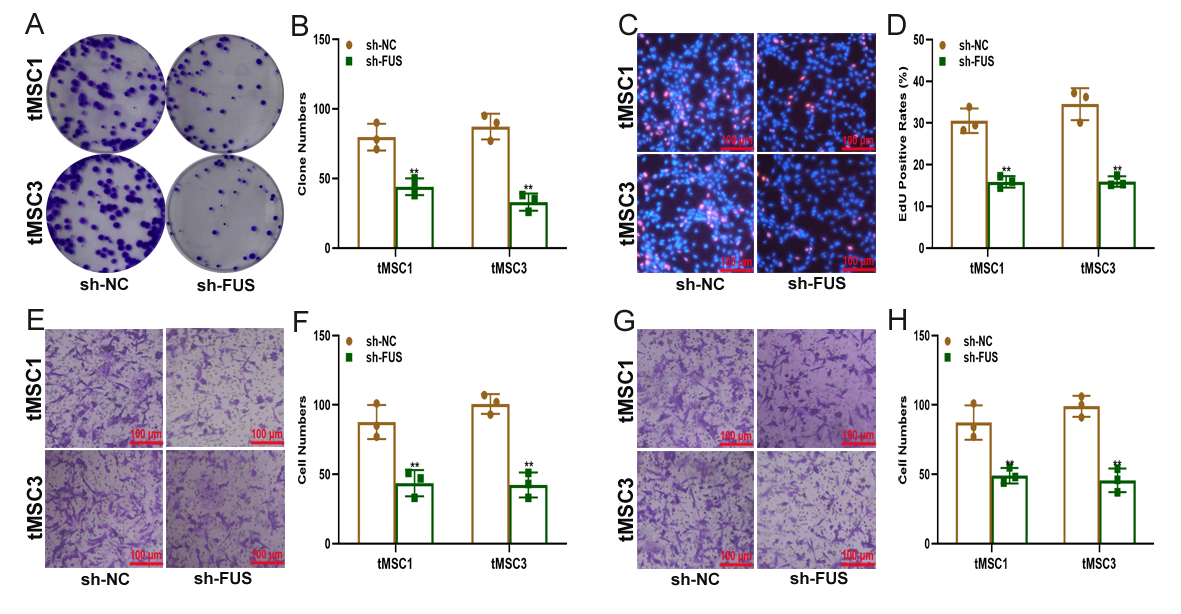
**Figure S1**

**Figure S1** The role of FUS in the proliferation, migration, and invasion of tMSC1 and tMSC3 cells.


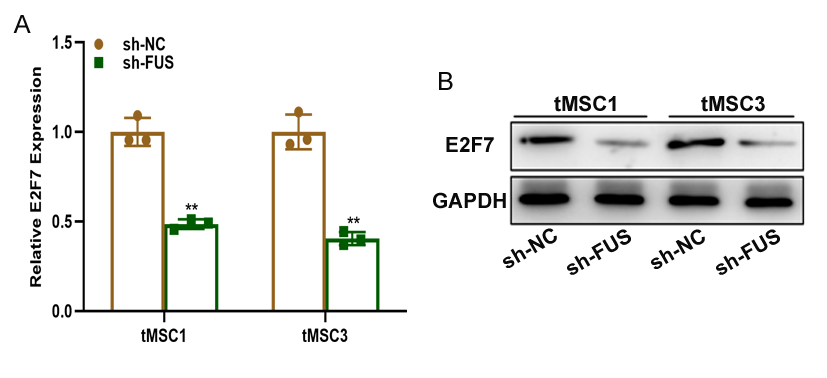
**Figure S2**

**Figure S2** qRT-PCR and Western blotting showed that downregulation of FUS could decrease the expression of E2F7


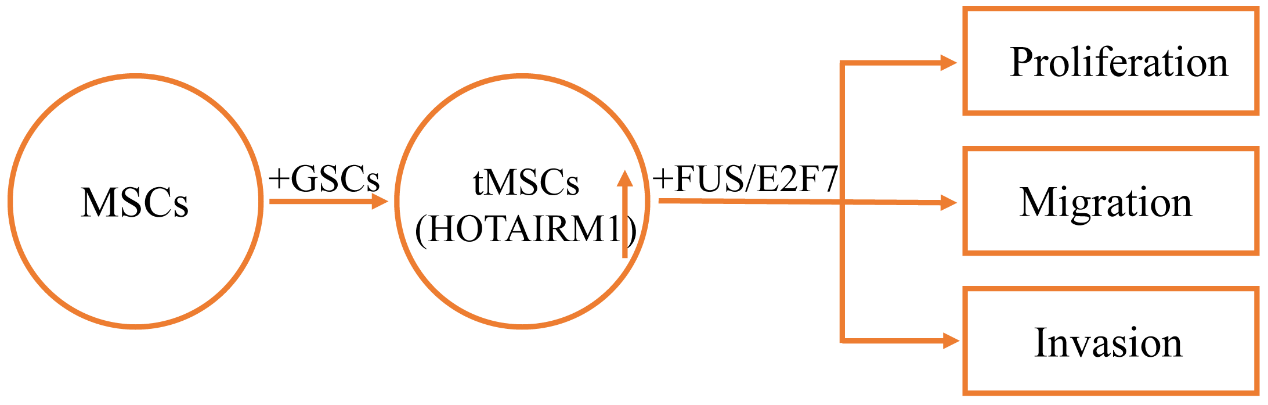
**Figure S3**

**Figure S3** The schematic overview of the HOTAIRM1/FUS/E2F7 in the malignant transformation.
